# *“Everything kind of revolves around technology”:* a qualitative exploration of families’ screen use experiences, and intervention suggestions

**DOI:** 10.1186/s12889-022-14007-w

**Published:** 2022-08-23

**Authors:** Lauren Arundell, Laura Gould, Nicola D. Ridgers, Ana Maria Contardo Ayala, Katherine L. Downing, Jo Salmon, Anna Timperio, Jenny Veitch

**Affiliations:** 1 Institute for Physical Activity and Nutrition, School of Exercise and Nutrition Sciences, Deakin University, Geelong, Australia; 2 Alliance for Research in Exercise, Nutrition and Activity (ARENA), Allied Health & Human Performance, University of South Australia, Adelaide, South Australia Australia

**Keywords:** Screen time, Family, Social interaction, Cyber safety, COVID-19, Interview, mHealth, Online intervention

## Abstract

**Background:**

Managing children’s screen time is challenging for most families. Interventions have had limited success in reducing screen time, potentially due to a lack of understanding of the experiences, needs and recommendations of families. This study aimed to 1) understand the screen time experiences of families, particularly during COVID-19 lockdowns; and 2) explore parent and child suggestions for the design, components, and content of a screen time management program.

**Methods:**

Parents and children from 30 families living in Victoria, Australia completed a semi-structured interview (63 interviews) via Zoom in October–November 2021. Parents were _m_aged 40.8 (± 8.9) years and predominantly female (90%). Children were _m_aged 11.4 (± 2.4) years and 47% female. The interviews were audio recorded, transcribed verbatim and analysed using inductive thematic analysis combined with a summative content analysis approach.

**Results:**

Three themes under Aim 1 emerged. Theme 1) ‘Screen time management experiences and practices’, including rules and strategies, challenges, and the impact of COVID-19 lockdowns. Theme 2) ‘Impact of screens on family interaction and communication’ including conflicts within the family, reduced face-to-face interactions, and negative impact on child’s behaviour and wellbeing. Theme 3) ‘Benefits of increased screen time due to COVID-19 lockdowns’ including continuation of social interactions, extracurricular activities, improved technology skills and using screens as a ‘babysitter’. Findings from Aim 2 suggest that families want a screen time management program delivered online to parents and children, which includes static and interactive content that incorporates health information, alternative activities, cyber-safety information, tips for goal setting and rewards, screen monitoring tools, links to reputable information, and parent social connections. Reminders via text message or through the online platform would help maintain engagement in the program.

**Conclusions:**

Families are experiencing challenges in managing the complex balance between the increased need for screens and the impact it has on the family. These findings provide valuable parent and child insights to assist in developing screen time management programs that are created with an understanding of the needs and challenges of families.

**Supplementary Information:**

The online version contains supplementary material available at 10.1186/s12889-022-14007-w.

## Introduction

The excessive time children spend using digital screen devices such as computers, phones, television and digital tablets is linked to poorer physical [1–3] and psycho-social health and wellbeing [4, 5], and is a major health concern of parents [6]. Guidelines from the World Health Organisation specify that children (aged 5–17) should limit daily recreational screen use to less than 2-h per day [7]. However, latest prevalence data (2011–2012) showed that 65% of Australian children aged 5–12 years exceed this, and guideline achievement declines further with increasing age [8]. The COVID-19 lockdown restrictions implemented in Australia and many countries worldwide saw the closure of schools, recreation and sporting facilities and the implementation of social distancing rules and restrictions. Consequently, schooling and social interactions moved online which led to increased time spent on screens for various reasons [9, 10]. Emerging evidence shows that elevated screen time during the COVID pandemic was associated with poorer levels of physical activity and sleep [11] and poorer mental health and greater perceived stress [12] amongst youth. Understanding the context and experiences of families and screen use in the home during lockdowns has received little research focus to date; however, parents' and young people's perceptions of screen use at home was a research focus prior to COVID-19.

In a qualitative study exploring engagement with contemporary screen devices pre-COVID lockdowns (2018) [13], a sample of Australian adolescents (*n* = 16, _m_aged 15.6 ± 2.4) acknowledged the complex influence of screen use on their lives including the potential benefits (e.g., more social interaction, screen time was calming) as well as the negative impacts (e.g., feelings of isolation and stress) [13]. This complexity is further highlighted in explorations of the perceptions of screen use amongst Canadian mother—pre-adolescents dyads (*n* = 91; pre-adolescents aged 10–13 years) [14]. For example, mothers recognised positive and negative socio-emotional effects of screen use for their children whereas children only noted positive socio-emotional effects [14]. Both mothers and pre-adolescents noted frustration around screen time rules but from different perspectives; mothers’ were frustrated when the child did not follow the rules, whereas the pre-adolescents were frustrated when their mother did not follow the rules [14]. Conflict around screen time management has been prevalent prior to COVID-19 lockdowns [6], but the dependence on screens for schooling, work, social interactions and leisure time [9] may have added another layer of complexity. Further it may have exacerbated the existing challenges with screen time management, the way in which families interact and communicate with one another and has the potential to raise new challenges and benefits. Families’ experiences with screen time during and following lockdowns, including management and the impact it has on conflict and interactions, are poorly understood. However, this information is crucial to underpin the development of effective intervention strategies targeting a reduction in screen time and the context in which they are delivered.

The past 20 years have seen unprecedented growth in the number of interventions targeting screen time amongst children [15]; however, a 2020 umbrella review of systematic reviews focusing on children’s sedentary behaviour and screen time interventions showed that, while effect sizes were significant, small changes of up to 17 min less screen time per day were noted [15]. However, most interventions to date have focused on traditional screens (e.g., TV viewing) without consideration of the current screen devices available [16], and they have not included parents and children in their development. It is important that new behavioural and digital strategies for families are informed by families [17, 18] to ensure programs are created with consideration of families’ needs and preferences, such as the delivery platform, content, and strategies for sustaining engagement and behaviour change [19, 20]. However, this has been rarely applied in the development of strategies targeting children’s screen time [17]. With the unknown lasting impacts of COVID-19 and associated lockdowns on children’s screen time, there has been a call for more qualitative research with families [21] to inform the redesign or development of new intervention strategies to minimise excessive screen use in the progression to ‘COVID-19 normal’. It is crucial to gain insights from both parents and children due to discordance in their reporting of screen time engagement during COVID-19 [22] and the differing impact screen time may have on familial interactions [14]. Further, ensuring end-user voice is included in the development of intervention programs can enhance the use, and effectiveness, of the program [23].

This study therefore aimed to 1) explore parents’ and children’s screen time experiences, particularly during the Victorian (Australia) COVID lockdown period; and 2) explore parent and child suggestions for the design, components, and content of a screen time management program.

## Methods

Families with a child aged 8–16 years, living in Victoria, Australia were invited to participate in parent and child interviews as part of the Healthy Happy Homes study via paid advertisements on social media (Facebook, Twitter, and Instagram). Interested participants clicked on the advertisement link which took them to the study information page on Qualtrics. This page contained the Plain Language Statement which they could download and save, and a consent form where they were asked to provide digital consent (via a tick box) for themselves (the parent/guardian) and their child to participate. Information including residential postcode, parent’s name, email, and date of birth, and child’s name, date of birth and current school year were also provided. The study team then contacted the parent to confirm eligibility and arrange a suitable time for the parent and child interviews. Given the broad aims of the study warranting a larger sample [24, 25] it was anticipated that 60 interviews from 30 parent–child dyads would be sufficient for reaching data saturation and was therefore the recruitment target. Recruitment strategies aimed to recruit *n* = 15 parent–child (8-12 years) and *n* = 15 parent-adolescent (13–16 years) dyads. Ethical approval was obtained from the Deakin University Human Ethics Advisory Group, Health (HEAG-H 139–2019).

The interviews were conducted from 2^nd^ September to 8^th^ October 2021 during which time Melbourne, Victoria was experiencing its sixth lockdown to curb the spread of COVID-19. At the end of this lockdown (21^st^ October 2021), metropolitan Melbourne residents had experienced a total of 262 days of lockdown since March 2020. During this time, ‘stay at home’ orders were in place whereby the only permitted reasons to leave home were to shop for necessities, care and care giving, exercise (with varying restrictions on the duration and number of people), authorised work and study, and to be vaccinated. Schools, workplaces, and recreation facilities (e.g., parks, skate parks) were closed and masks were required to be worn at all times outside of the home (though schools were open for children of essential workers, e.g., nurses). At times, individuals were to stay within 5 km from their home and there was a curfew in place from 9 pm to 5am. Regional and rural Victoria experienced similar lockdowns over 2020/2021 but these were generally with fewer restrictions and for shorter durations. As such, at the start of data collection regional Victoria were in their seventh lockdown, with large regional towns moving in and out of short (7–14 days) lockdowns over the following two months.

Semi-structured interviews were conducted via Zoom. Parents and children were interviewed separately and sequentially, however if desired the parent was able to be present during the child interview. The interviews had two focus areas aligned with the aims of the study. The first was to explore parents’ and children’s current experiences with screen time management including any rules or restrictions in place, any impact of screen time on familial interactions and communications benefits, and any challenges of screen time due to COVID-19 lockdowns. The second focus was to gain suggestions for the development of a screen time management program. Specifically, participants were asked what information, strategies or content they would like in a program, whether it should involve the parent as well as the child, and how best to maintain engagement in a program. Parents were also asked about their preferred delivery platform (e.g., website, apps, etc.) and delivery mode (e.g., interactive versus static content, information shared at once or over time). The parent and child interview schedules were pilot tested with two families (two parents and two children) prior to data collection, with question wording adapted as needed (see Additional File 1 for interview schedules). All participants received a $25 e-voucher (Target) as compensation for their time. This study was conducted and reported in line with the Consolidated Criteria for Reporting Qualitative Research (COREQ; see Additional File 2) [26].

All interviews were audio recorded and transcribed verbatim. As the interview questions were created with consideration of previous literature, the qualitative data were analysed using an inductive thematic analysis (Aim 1) [27] combined with a summative content analysis approach (Aim 2) [28]. Thematic analysis is a method of identifying, analysing, organising, describing and reporting themes from qualitative data [27]. Its flexibility allows for the identification of patterns or themes in large datasets and for the researcher to interpret the importance of the theme without consideration of its frequency or size within the data [27, 29]. It enables the exploration of perspectives from different participants (e.g., parents and children) [29]. Braun and Clarke’s [27] six-phases of thematic analysis were followed in this study. Two authors (LA and LG) read all interview transcripts to familiarise themselves with the data (phase 1), created the initial coding framework based upon the interview foci (phase 2), and then searched for themes (phase 3). Both authors used NVivo qualitative data software (version 12.6) to help code the data within this framework while allowing for codes to form inductively. Following, they met to discuss, compare, review, and verify codes and themes (phase 4). Concurrently, both authors grouped the codes into sub-themes within themes, and together defined and named the themes (phase 5), which aligned with the first part of the interview’s focus (Aim 1) [27]. Content from responses to the second part of the interview (Aim 2) were grouped to identify frequently occurring preferences/suggestions for the development of a screen time management program by both authors. These were grouped according to the component of the program they related to (e.g., program delivery, program audience etc.). The results were discussed and confirmed with the research team (phase 6).

## Results

The final sample comprised 30 families. In one family, both parents were present for the parent interview and their twin children completed separate interviews, and in another family two parents and two children were interviewed separately, resulting in a total of 63 interviews (31 parents, 32 children). Parents were on average 40.8 (± 8.9) years and predominantly female (90%) and the children were on average 11.4 (± 2.4) years and 47% were female. Twenty-five families lived in metropolitan Melbourne and five lived in regional/rural areas. The combined parent and child interviews lasted on average 38 min, 13 s per family. Findings were combined for the child and adolescents dyads where similar themes emerged, and any differences according to age are described in the results.

There were three interview themes from Aim 1, which are shown in Table 1 and described in detail with relevant quotes below. These included families screen time management experiences, the impact of screen time on family interaction and communication, and the benefits of increased screen time due to lockdown.

**Table 1:** Key themes from the qualitative interviews with parents and children (Aim 1)

Theme 1: Screen time management: experiences and practices	
1.1 **Screen time rules and strategies** - Rules about timing of screen use (P, C) - Rules about location of screen use (P, C) - Stricter rules on the weekends (P, C) - Rules about monitoring/cyber-safety (P, C)	1.2 **Challenges** - Addictive nature of screens (P, C) - Child breaking rules (P, C) - Parenting style misalignment
1.3 **Impact of COVID-19 lockdowns** - Parents relaxed screen time rules (P) - Remote learning increased screen time requirements (P, C) - Parent work commitments, reduced time to supervise (P) - Screens enabled social connections to be maintained (P) - Lack of physical activity/other activities to do (P, C) - Child ‘over’ screens (P, C)	1.4 **No management required** - No rules (P) - Lack of interest/ ‘not a screen kid’ (P) - Child self-manages/regulates time (P, C)
**Theme 2: Impact of screens on family interaction and communication**	
2.1 **Conflicts within family** - Increased conflict with family and siblings (P, C) - Increased conflict between parents’ management and rules (P)	2.2 **Reduced face-to-face communication** - Less face to face talking, less down time (P, C) - More isolated screen time (C) - Increased screen use among parents (C)
2.3 **Negative impact on child’s behaviour and wellbeing** - Negative impact on sleep and behaviour (P, C) - Negative impact on mood, emotional effects (P, C)	2.4 **Positive/no impact on family interaction and communication** - No impact on family interactions (P, C) - Greater interaction between children (P)
**Theme 3: Benefits of increased screen time due to COVID-19 lockdowns**	
**Helped maintain social interactions** - Social interactions with friends could continue (P, C) - Additional interactions with siblings (P, C)	3.2 **Facilitated the continuation of activities** - Schooling could continue (P, C) - Extracurricular activities could continue (P)
3.3: **Improved technology skills for parent and child** (P)	3.4: **Screens used as a ‘babysitter’** (P)

*P* Identified from parent interviews, *C* Identified from child interviews

### Theme 1: Screen time management experiences

#### Sub-theme 1.1 Screen time rules and strategies

Most parents and children had rules around the timing of the child’s screen use. These related to the length of time the child could use screens for, the time of day they could use them, and where the screens could be used in the home with many parents restricting use in bedrooms overnight and during mealtimes. Differences in rules between weekdays and weekend days were noted by many parents, with parents commonly affording children more screen time on weekends. Many parents noted they used warnings, timers, or settings on the device to enforce the time limits.

*“They don’t have them at mealtimes, or in the toilet [or] the bathroom. Definitely not while eating, and mealtimes – that’s family time.”* (Parent aged 34, female; child aged 10, male)

Many parents and some children reported rules to ensure cyber-safety. These related to not being allowed to talk to people they do not know, having someone they know online with them, and not revealing anything about where they live.

*“Anything that involves online communication, the rules are that you are not to talk to anyone that you have not physically ever met. And that both brothers should be logged on and talking, especially with the younger one,”* (Parent aged 43, female; child aged 13, male)

#### Sub-theme 1.2 Challenges

Most parents and children spoke about challenges with screen time management. Specifically, most noted that the addictive nature of screens made managing both their own and their child’s use difficult. Many parents explained how their child would often use the screens without realising the duration of use, and that their screen use would take over other aspects of their life, for example, excessive conversations about gaming, and wanting to stop other activities to return to playing. Children often explained how they felt it was difficult to turn off the screens and felt addicted to it.

*“We had to ban Roblox for ages because [brother] wouldn’t go to the toilet. He would wet himself playing games because he didn’t want to die in the game.”* (Parent aged 48, female; child aged 9, female)

*“If they tell me that I can’t go on it every day of the week, then I’ll probably still go on it because I’m kind of addicted to it.”* (Child aged 9, female)

Many parents noted challenges associated with their child breaking the screen time rules they had in place. Children would often ignore or not abide by the rules set by the parents (e.g., sneak devices into their bedrooms) as well as the tools built into devices to manage usage (e.g., silent access timers). Some parents also spoke about a misalignment between their own and their parenting partners’ screen time rules and expectations.

*"When our timers go off, sometimes we just stay on for longer than we’re meant to. We just kind of say something like, ‘I’m finishing this video.’ And then like start another video and … kind of lose track and keep going and going and going.”* (Child aged 12, female)

*“It’s a free for all when my husband is around with technology. Different rules. That’s his rules.”* (Parent aged 35, female; child aged 9, female)

#### Sub-theme 1.3 Impact of COVID-19 lockdown

Most parents acknowledged that COVID-19 related lockdown restrictions had impacted their child’s screen time and the management of these behaviours. Many parents spoke about the difficulties trying to supervise their child’s screen time while completing their own work. Parents and children spoke about the necessary increased use of screens for remote learning, with many noting that the child would get distracted by the functionality of the program (e.g., change fonts, colours, etc.) which would extend the task, and subsequently the screen time.

*"I cannot physically watch three children’s stuff that happens during school hours. I know everything they're watching outside school hours, but there’s no humanly possible way - with three kids in one room and two adults working from home, and all the meetings are at the exact same time for the kids...it's really hard"* (Parent aged 41, female; child aged 10, female)

Many parents acknowledged that they had relaxed their screen time rules due to COVID-19 lockdowns, but few children identified that the rules had changed due to lockdown. Most parents and children also noted there were different rules for different screen devices, with schooling and social screen use less restricted. Many families also described that they now used screens more for family time, for example family movie night.

*“They’re not allowed TV or iPads after six generally. It used to be five. And once we had dinner, we didn’t have TV on. Now on a Saturday, since COVID came along, we have movie night on a Saturday night, which means they’ll often watch a couple of movies.”* (Parent aged 40, female of child aged 11, female)

Parents and children spoke about there being little else to do during lockdown, and therefore they both defaulted to using screens. The lack of in-person school, sports, extracurricular activities and social outings during lockdown meant this time was often spent using a screen. Parents also encouraged breaks from the screens during lockdown, with many suggesting their child went outside or was active.

*“Because of lockdown, I’m not as busy. Normally I’d have school, so I wouldn’t be on it as much, and then I’d have sports after school, or doing the musicals, so I would be occupied. But during lockdown there’s not much else to do, so everything kind of revolves around technology.”* (Child aged 14, female)

A few parents and children discussed how the increased need to use screens for schooling resulted in them no longer wanting to use screens outside of school times.

*“He’s sick of it [screens]. He’s sick of doing his work and he’s sick of watching his teacher on there, and he’s sick of his WebEx’s and his Zoom meetings … he’s sick of it.”* (Parent aged 40, female; child aged 8, male)

#### Sub-theme 1.4 No management required

A few parents recognised that their child had little interest in screens and so there was little management required. Their child would prefer to engage in other activities, often physically active games, instead of using screens. Some parents and children also spoke about allowing their child to learn to manage their own screen time. No children and only a few parents reported having few or no rules around screen time, believing that their child would oppose strict guidelines, or their child was able to self-regulate their screen time.

*“I’m of the opinion that the children need to learn their own boundaries. Basically, I let them manage their own [screen time]. I’m of the belief that if I’m managing them on their screen time, they’re never going to learn how to do it as adults.”* (Parent aged 49 female, parent of child aged 16 male)

### Theme 2: Impact of screens on family interaction and communication

#### Sub-theme 2.1 Conflicts within family

Almost all parents and children spoke about the conflicts between family members because of screen use. This included conflict between parents and children, between siblings, and between parents where their screen time management styles or expectations differed.

“*We shout at each other because I won’t want to get off, and we might shout at [mum] because me and my dad will be playing the PlayStation and we’re going to do a tournament at five o’clock, so mum will complain because we normally have dinner at five o’clock.”* (Child aged 8, male)

#### Sub-theme 2.2 Reduced face-to-face communication

Most parents and children recognised that screen time reduced face-to-face communication between family members and resulted in less time spent together. Many children spoke about how screens were frequently used in isolation from others. Children also identified that their parents were often on screens which reduced communication between parents and between child and parent.

*“I noticed the other night we’re all on our phones sitting there, the TV is on, we’re not watching it, not having a conversation. You’re losing that conversation and interaction.”* (Parent aged 55, female; child aged 14, female)

#### Sub-theme 2.3 Negative impact on behaviour and wellbeing

Many parents and some children spoke about how the child’s screen time increased tiredness as the child would be on their screens instead of sleeping. Parents also discussed the impact of screen use on the child’s mood and behaviour noting that this would influence how the family would interact, often leading to conflict and tension within the home.

*“When she’s tired when she’s had a lot of screentime, as I said that little cyber demon comes out, it creates more arguments.”* (Parent aged 43, female; child aged 10, female)

*“I feel like being on the computer for the entire day kind of gets to me, and I don’t really sleep very well, and sometimes I get a bit emotional, sad for reasons I don’t really know.”* (Child aged 12, male)

#### Sub-theme 2.4 Positive/no impact on family interaction and communication

Many parents, but no children, discussed how screen time resulted in greater interaction between siblings. This tended to be when they played the same game together. Some parents felt that screen time did not negatively impact their interaction or communication as a family. This seemed particularly evident among families with older children who were able to self-manage their screen time. Few children felt that that there was no impact on family interaction and communication.

*“Sometimes [the kids] go to get Minecraft together and they’ll actually enter each other’s worlds and then they will interact relatively well during that time.”* (Parent aged 40, female; child aged 11, female)

### Theme 3: Benefits of increased screen time due to COVID-19 lockdowns

#### Sub-theme 3.1 Helped maintain social interactions

The most commonly mentioned benefit of screen time during COVID lockdowns was that it helped children to maintain their social interactions in the absence of school, extracurricular, and social activities. Parents also spoke about screen time facilitating interactions between family where they were playing similar games, watching shows together, and forming connections.

*“Not being able to hang out at the beach with her friends, not being able to just do all the things that 14-year-olds should probably be doing. It’s nice that she can at least use social media, which I never thought I would say, to connect socially”.* (Parent age not provided, female; child aged 9, female)

#### Sub-theme 3.2 Facilitated the continuation of activities

Parents and children discussed the benefit of being able to continue with schooling via screens during lockdown. Many parents also mentioned how extracurricular activities could continue online during lockdown.

*“There’s been karate [online] and my daughter has a swimming program that she can attend. So that connection has stayed there.”* (Parent aged 40, female; child aged 11, female)

#### Sub-theme 3.3 Improved technology skills for parent and child

Some parents identified that the increased screen use during lockdown had improved technology skills of the parents and child. This included program-specific skills (e.g., Google for information), spelling and grammar (e.g., via autocorrect) as well as the vocabulary and abbreviations children use online.

*“One of the benefits is it’s so regular now, it’s become easier to do. For me it’s actually easier to use technology than what it was before COVID.”* (Parent aged 35, female; child aged 9, female)

#### Sub-theme 3.4 Screens used as a babysitter

When discussing the benefits of screen time during lockdown, some parents appreciated that it allowed their child to be occupied and they did not need to supervise them. Some parents recognised that they used screen time as a ‘babysitter’ and were more lenient with screen time to give themselves a break, allow them to complete their own tasks or sleep-in.

*“I want to limit screen time a lot more, but I think out of my own sanity sometimes I'm just like, “Take your tablets and go. Just get out. Leave me alone”.”* (Parent aged 39, female; child aged 9, female)

### Suggestions for programs to help manage screen time in the home environment

**Fig. 1:**
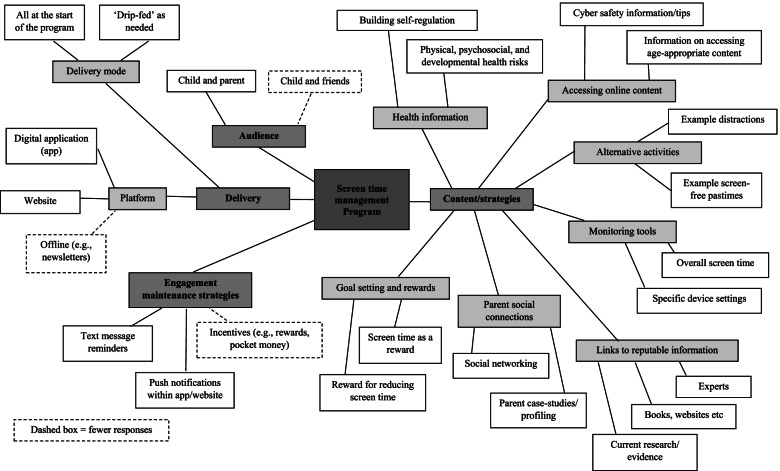
Characteristics of a screen time management program identified by families

Parent and child suggestions for intervention programsto help manage children’s screen time are shown in Fig. 1 and described below (for brevity quotes have not been included).

#### Program delivery

Most parents reported that they would like a program to be delivered online either via an app or website platform. Only a few suggested it would also be useful to have ‘offline’ supportive materials (e.g., posted newsletters). There was a mixture of responses regarding frequency of delivery of program content, with many saying they would like to receive all information and content at once so they could read it, whereas others noted they would prefer to have the program ‘drip-fed’ with new information provided regularly.

#### Program audience

Most parents wanted a screen time management program to be for both the parent and child. Many wanted their child to be involved in any decision making incorporated into the program believing this may result in greater compliance, while others felt that being involved would help reduce their own screen use. Many children wanted their parents to be involved and a few also thought their friends should be involved.

#### Program content

Parents and children reported a variety of strategies or content that would be useful to their family. Parents and children noted they wanted a program to include information on the physical, psycho-social, and developmental health risks of screen time. Many parents mentioned that it would be important for information to be provided in child-friendly language so they could easily understand the risks. Incorporating information about cyber safety was raised by many parents and some children as important. Parents noted including information about not interacting with strangers, accessing only suitable and age-appropriate content, and potential settings that families could use on their devices to support this. Children’s cyber safety suggestions were based around social media and what was or was not suitable to post online.

Monitoring tools such as timer options and device settings that limited screen time were suggested by both parents and children as an important strategy to include in the program. Parents suggested that these were important for overall screen time and specific devices and were particularly important amongst parents who acknowledged they had poor technological skills or used other devices (e.g., Apple vs Android). Parents and children spoke of using rewards within the program. Some parents suggested that the child receive a reward when they reduce their screen time, whereas children also suggested that screen time could be used as the reward. Incorporating challenges or games were suggested as ways to provide variety and motivation to reduce screen time. Many parents also noted that these would provide an opportunity for the whole family to be involved, promote healthy competition and bonding. The challenges and games were considered particularly relevant and appealing for younger children.

In addition, many parents reported that they would like assistance in setting screen time goals and suggestions for distractions or alternative pastimes/activities to screen use. Many parents reported wanting information and strategies to assist their child with self-regulation of their screen time, and the consequences of their behaviours. The benefit of hearing from other’s experiences was also noted as an important component for parents with suggestions for inclusion of social networking opportunities or parent profiling. Many parents also wanted the program to include links to reputable information or websites that contained current research about screen time, suitable apps and games, and effective management strategies.

#### Maintaining program engagement

Most parents believed that reminders about the program would help maintain their engagement; however, there were many differing preferences for how the reminders should be delivered. For example, some parents suggested text messages whereas others thought this would be an annoyance, and others thought push notifications embedded within an app/web-based program would be most effective. Many children felt that their engagement could be maintained through incentives (e.g., pocket money, rewards) and if the program was updated regularly to keep it varied and new.

## Discussion

This study provides an in-depth understanding of families’ experiences managing screen time, the impact that screen time has on family interactions and communications, and provides invaluable information for the development of programs to help manage screen use. The findings highlight the numerous and varied challenges that families experience when trying to manage screen time, particularly under COVID-19 lockdown restrictions. Quantitative data has shown that children’s and parents’ screen use increased during lockdown in Australia [9] and globally [10]. The current findings provide insights into the current screen time context within homes and suggestions for development of a program to help families manage screen time. The findings should be considered when developing or updating strategies targeting pervasive screen time.

Most families in the current study had existing rules and strategies to manage screen use, and the presence of rules has consistently been associated with lower screen time amongst children [16, 30, 31]. However, during lockdowns parents noted that many of these rules and strategies were relaxed to facilitate the child’s schooling, social interactions, and extracurricular activities as well as the parents’ work commitments. While these activities are beneficial for children’s academic, physical and social outcomes [32–35] sustained engagement via a screen may negatively impact the child’s behaviour and wellbeing. It is important to understand how families’ rules and strategies continue to change as lockdowns and restrictions ease and the necessity to use screens for schooling, socialisation and activities may change. Parents may need additional support to reinstate previous rules or develop new rules with consideration of how their child now uses screens as restrictions change over time. This information will be important content to include in a screen time management program for families.

The change in use and acceptability of screens during COVID lockdowns was evident in the current study, with families reporting that screens were at times used for ‘family time’ (e.g., families enjoying a movie night or siblings co-participating in electronic games). However, most families acknowledged the negative impacts of screen time on the way their family interacted (or not) with each other and the conflict it would cause between siblings and/or parents. Most families in the current study recognised how screen time negatively impacted their child’s behaviour and mood, which aligns with previous quantitative research [36]. This association may potentially be due to lower sleep duration [37, 38] which was also noted by parents in the current study as a detrimental impact of screen time. A major concern for parents prior to COVID-19 lockdowns was the conflict it caused [6] and the impact that screen time may have on their child’s wellbeing [39]. It is therefore important to update our understanding of the impact that typically beneficial activities (e.g., schooling, social interaction, activities) performed via screens have on children’s health and wellbeing. Further, as COVID-19 related lockdown restrictions are eased, and families move towards living with ‘COVID-normal' there may be a continued perfusion of screens in daily life via blended work and learning. The challenge for researchers and families is ensuring this evolving context, after increased exposure, is considered when creating strategies for screen use management.

This study generated important insights into the needs and preferences of parents and children that should be considered in the development of a screen time management program for families. Despite the purpose of such programs to manage screen time (reduce time spent online), families are seeking online delivery of information due to the ease of access, ability for large amounts of information to be accessible to families, and available when and how they require it. It would also enable scale up to a wider audience. The content parents wanted in the program included static information (e.g., the health risks) but also practical help (strategies, tips, advice, and examples from other parents). Importantly, many of the suggestions for the program made by parents align with the characteristics and behaviour change techniques of screen time interventions that have previously been shown to be effective amongst children (0–18 years; e.g., the provision of educational materials, goal setting, planning and monitoring, role-modelling and behaviour substitution [16, 20, 40, 41]) but are lacking in most commercial apps targeting health behaviours (e.g., diet, physical activity and sedentary behaviour) [42]. Further, many of these strategies are also elements of ‘gamification’ within Schmidt-Kraepelin’s 2018 Taxonomy of Gamification Concepts for Health Apps [43], including rewards, goal-setting and reinforcement, which have been shown to support behaviour change while being fun and engaging [44]. Therefore, a screen time management program designed based on the findings of this study may be engaging and effective and requires development and testing.

Ongoing engagement in behaviour change programs remains a challenge for interventions. The suggested use of digital reminders (via SMS, app notifications or website) or delivery of intervention content may assist with maintaining engagement. Previously, text message interventions have shown promise in reducing screen time [45], and they may also serve as supportive reminders. As there was some variability in the suggested platform, content delivery and reminder methods proposed by families, multiple options may need to be available within a program and it may be important to allow the ability to personalise components to suit families (e.g., they can select how they receive reminders). Similarly, such personalisation or tailoring of health messages have been shown to be more engaging and effective than generic messages in behaviour change interventions [46] and therefore warrant further investigation in managing screen time.

The limitations of the current study include that the participants were from one state of Australia (Victoria), and the metropolitan area experienced one of the longest COVID-19 lockdown restrictions in the world. The screen time management experiences, challenges and opportunities described may be different to families in other states and countries. However, the interviews also explored usual screen time experiences providing valuable information from both COVID lockdown and non-COVID lockdown contexts. The strengths include its contribution to limited qualitative research about screen time management experiences and the inclusion of both parents and children. Findings provide insights that can be used by researchers and practitioners when aiming to manage screen time. Further, it provides an important step to inform the development of interventions aimed at managing children’s screen time. The findings will enable the important integration of behaviour change science and mHealth design thinking to develop more effective interventions [47]. By infusing these findings, interventions can be created with an understanding of the end-users’ needs, challenges and influencing factors so they achieve sustained engagement.

## Conclusion

This study highlights that families experience many challenges with managing screen time. This is particularly evidenced in their attempt to balance the increased need for screen use and the impact screen time has on familial communications and interactions, child behaviour, mood, and sleep, which has been exacerbated by COVID-19 lockdown restrictions. These findings provide valuable parent and child insights to assist in developing screen time management programs that are created for sustained behaviour change with an understanding of the needs and challenges of families.

## Supplementary Information

**Additional file 1.** Heathy Happy Homes Interview schedule.

**Additional file 2.** Consolidated criteria for reporting qualitative studies (COREQ) checklist.

## Data Availability

The data cannot be made publicly available due to ethical requirements but may be shared on reasonable request from the corresponding author.

## References

[1] Sanders T, Parker PD, del Pozo-Cruz B, Noetel M, Lonsdale C (2019). Type of screen time moderates effects on outcomes in 4013 children: evidence from the Longitudinal Study of Australian Children. Int J Behav Nutr Phys Act.

[2] Gabel L, Ridgers ND, Della Gatta PA, Arundell L, Cerin E, Robinson S (2016). Associations of sedentary time patterns and TV viewing time with inflammatory and endothelial function biomarkers in children. Pediatr Obes.

[3] Trinh L, Wong B, Faulkner GE (2015). The Independent and Interactive Associations of Screen Time and Physical Activity on Mental Health, School Connectedness and Academic Achievement among a Population-Based Sample of Youth. J Can Acad Child Adolesc Psychiatry.

[4] Mistry KB, Minkovitz CS, Strobino DM, Borzekowski DLG (2007). Children's Television Exposure and Behavioral and Social Outcomes at 5.5 Years: Does Timing of Exposure Matter?. Pediatr.

[5] Bize R, Johnson JA, Plotnikoff RC (2007). Physical activity level and health-related quality of life in the general adult population: a systematic review. Prev Med.

[6] Rhodes A. Screen time and kids: What's happening in our homes? The Royal Children's Hospital, Australian Child Health Poll; 2017. Available at: www.rchpoll.org.au/. Accessed 8 Aug 2021.

[7] World Health Organisation (2020). WHO guidelines on physical activity and sedentary behaviour.

[8] Australian Institute of Health and Welfare. Physical activity across the life stages. Cat. no. PHE 225. Canberra, Australia; 2018. Available at https://www.aihw.gov.au/reports/physical-activity/physical-activity-across-the-life-stages/contents/summary. Accessed 8 Aug 2021.

[9] Arundell L, Veitch J, Sahlqvist S, Uddin R, Ridgers ND, Salmon J (2021). Changes in Families’ Leisure, Educational/Work and Social Screen Time Behaviours before and during COVID-19 in Australia: Findings from the Our Life at Home Study. Int J Env Res Public Health.

[10] Paterson DC, Ramage K, Moore SA, Riazi N, Tremblay MS, Faulkner G (2021). Exploring the impact of COVID-19 on the movement behaviors of children and youth: A scoping review of evidence after the first year. J Sport Health Sci.

[11] Moitra P, Madan J (2022). Impact of screen time during COVID-19 on eating habits, physical activity, sleep, and depression symptoms: A cross-sectional study in Indian adolescents. PLoS ONE.

[12] Nagata JM, Cortez CA, Cattle CJ, Ganson KT, Iyer P, Bibbins-Domingo K (2022). Screen Time Use Among US Adolescents During the COVID-19 Pandemic: Findings From the Adolescent Brain Cognitive Development (ABCD) Study. JAMA Pediatr.

[13] Thomas G, Bennie JA, De Cocker K, Biddle SJH (2021). Exploring contemporary screen time in Australian adolescents: A qualitative study. Health Promot J Austr.

[14] Francis K, Scholten H, Granic I, Lougheed J, Hollenstein T (2021). Insights about Screen-Use Conflict from Discussions between Mothers and Pre-Adolescents: A Thematic Analysis. Int J Env Res Public Health.

[15] Nguyen P, Le LK-D, Nguyen D, Gao L, Dunstan DW, Moodie M (2020). The effectiveness of sedentary behaviour interventions on sitting time and screen time in children and adults: an umbrella review of systematic reviews. Int J Behav Nutr Phys Act..

[16] dos Santos PC, Barbosa Filho VC, da Silva JA, Bandeira AdS, Minatto G, da Silva KS. What Works in Sedentary Behavior Interventions for Youth: A Review of Reviews. Adolesc Res Rev. 2019;4(3):267–92.

[17] Altenburg TM, Holthe JK-v, Chinapaw MJM (2016). Effectiveness of intervention strategies exclusively targeting reductions in children's sedentary time: a systematic review of the literature. Int J Behav Nutr Phys Act..

[18] Ball K, Cleland V, Dollman J, Turrell G. Action area 7: Disadvantaged populations. In: Blueprint for an active Australia. 2nd edn. Melbourne: National Heart Foundation of Australia. 2014. Available at www.heartfoundation.org.au/images/uploads/publications/Blueprint-for-an-active-Australia-second-edition. Accessed 8 Aug 2021.

[19] Marsh S, Foley LS, Wilks DC, Maddison R (2014). Family-based interventions for reducing sedentary time in youth: a systematic review of randomized controlled trials. Obes Rev.

[20] Biddle SJH, Petrolini I, Pearson N (2014). Interventions designed to reduce sedentary behaviours in young people: a review of reviews. Br J Sports Med.

[21] Korhonen L (2021). The good, the bad and the ugly of children´s screen time during the COVID-19 pandemic. Acta paediatrica (Oslo, Norway: 1992).

[22] Nagata JM, Cortez CA, Iyer P, Ganson KT, Chu J, Conroy AA (2022). Parent-Adolescent Discrepancies in Adolescent Recreational Screen Time Reporting During the Coronavirus Disease 2019 Pandemic. Acad Pediatr.

[23] Thabrew H, Fleming T, Hetrick S, Merry S. Co-design of eHealth Interventions With Children and Young People. Front Psychiatry. 2018;9:481. 10.3389/fpsyt.2018.00481 PMC6200840 30405450

[24] Braun V, Clarke V (2022). Conceptual and design thinking for thematic analysis. Qualitative Psychology.

[25] Malterud K, Siersma VD, Guassora AD (2016). Sample Size in Qualitative Interview Studies: Guided by Information Power. Qual Health Res.

[26] Tong A, Sainsbury P, Craig J (2007). Consolidated criteria for reporting qualitative research (COREQ): a 32-item checklist for interviews and focus groups. Int J Qual Health Care.

[27] Braun V, Clarke V (2006). Using thematic analysis in psychology. Qual Res Psychol.

[28] Hsieh HF, Shannon SE (2005). Three approaches to qualitative content analysis. Qual Health Res.

[29] Nowell LS, Norris JM, White DE, Moules NJ (2017). Thematic Analysis: Striving to Meet the Trustworthiness Criteria. Int J Qual Methods.

[30] Maniccia DM, Davison KK, Marshall SJ, Manganello JA, Dennison BA (2011). A meta-analysis of interventions that target children's screen time for reduction. Pediatrics.

[31] Gingold JA, Simon AE, Schoendorf KC (2014). Excess screen time in US children: association with family rules and alternative activities. Clin Pediatr (Phila).

[32] Morrissey TW, Hutchison L, Winsler A (2014). Family income, school attendance, and academic achievement in elementary school. Dev Psychol.

[33] Logan K, Cuff S (2019). Organized Sports for Children, Preadolescents, and Adolescents. Pediatr.

[34] Bjørnarå HB, Westergren T, Sejersted E, Torstveit MK, Hansen BH, Berntsen S (2021). Does organized sports participation in childhood and adolescence positively influence health? A review of reviews. Prev Med Rep.

[35] Eime RM, Young JA, Harvey JT, Charity MJ, Payne WR (2013). A systematic review of the psychological and social benefits of participation in sport for children and adolescents: informing development of a conceptual model of health through sport. Int J Behav Nutr Phys Act.

[36] Boers E, Afzali MH, Newton N, Conrod P. Association of Screen Time and Depression in Adolescence. JAMA pediatrics. 2019;173(9):853-9. 10.1001/jamapediatrics.2019.1759 PMC6632122 31305878

[37] Lemola S, Perkinson-Gloor N, Brand S, Dewald-Kaufmann JF, Grob A (2015). Adolescents’ Electronic Media Use at Night, Sleep Disturbance, and Depressive Symptoms in the Smartphone Age. J Youth Adolesc.

[38] Parent J, Sanders W, Forehand R (2016). Youth Screen Time and Behavioral Health Problems: The Role of Sleep Duration and Disturbances. J Dev Behav Pediatr.

[39] Arundell L, Parker K, Salmon J, Veitch J, Timperio A (2019). Informing Behaviour Change: What Sedentary Behaviours Do Families Perform at Home and How Can They Be Targeted?. Int J Env Res Public Health..

[40] Jones A, Armstrong B, Weaver RG, Parker H, von Klinggraeff L, Beets MW (2021). Identifying effective intervention strategies to reduce children’s screen time: a systematic review and meta-analysis. Int J Behav Nutr Phys Act.

[41] Lewis L, Povey R, Rose S, Cowap L, Semper H, Carey A (2021). What behavior change techniques are associated with effective interventions to reduce screen time in 0–5 year olds? A narrative systematic review. Prev Med Rep.

[42] Schoeppe S, Alley S, Rebar AL, Hayman M, Bray NA, Van Lippevelde W (2017). Apps to improve diet, physical activity and sedentary behaviour in children and adolescents: a review of quality, features and behaviour change techniques. Int J Behav Nutr Phys Act.

[43] Schmidt-Kraepelin M, Thiebes S, Tran MC, Sunyaev A, editors. What's in the Game? Developing a Taxonomy of Gamification Concepts for Health Apps. HICSS; 2018. 150.

[44] Cugelman B (2013). Gamification: what it is and why it matters to digital health behavior change developers. JMIR Serious Games.

[45] Ludwig K, Arthur R, Sculthorpe N, Fountain H, Buchan DS (2018). Text Messaging Interventions for Improvement in Physical Activity and Sedentary Behavior in Youth: Systematic Review. JMIR Mhealth Uhealth.

[46] Fjeldsoe BS, Marshall AL, Miller YD (2009). Behavior Change Interventions Delivered by Mobile Telephone Short-Message Service. Am J Prev Med.

[47] Voorheis P, Zhao A, Kuluski K, Pham Q, Scott T, Sztur P (2022). Integrating Behavioral Science and Design Thinking to Develop Mobile Health Interventions: Systematic Scoping Review. JMIR Mhealth Uhealth.

