# Supplementary material for: “Everything kind of revolves around technology”: a qualitative exploration of families’ screen use experiences, and intervention suggestions
Source: BMC Public Health. 2022 Aug 23;22:1606. doi: 10.1186/s12889-022-14007-w (PMC9398049; doi:10.1186/s12889-022-14007-w)
Supplement: Supplementary file 1 — Additional file 1. Heathy Happy Homes Interview schedule. [file 12889_2022_14007_MOESM1_ESM.docx]

**Additional File 1: Heathy Happy Homes Interview schedule**

**PARENT QUESTIONS**

- What are the main types of screens that are used in your home (by child and wider family)?
- Is managing your/your child’s screen time a challenge? Yes/No, prompt: why do you think that is?
- Do you have rules or strategies to help manage screen use in your home? *Example probing questions* no screens in the bedroom, usage/management controls (*Example probing questions* timers for use) for yourself or your child?
- With COVID-19 lockdown restrictions, have you/your child experienced any changes in screen time behaviours?
  - Are there any new challenges you/your child has experienced?
  - Are there any benefits you are experiencing due to this (Example probing questions efficiencies, social connections, online physical activity options?)?
- Does screen time impact how your family interacts and communicates with each other (*Example probing questions* leads to arguments/ more or less talking with each other)?
- Are there any other impacts of your/your child’s screen use?

We are wanting to develop effective strategies and programs to help manage screen time at home. To ensure these are as effective as possible we would love to have your input.

- - Firstly, can you tell us who and where you would (and have you) seek information or help to manage screen time.
  - What type of individual/group would you be most likely to seek info from and listen to? Example probing questions researchers, General Practitioner, websites, teachers/schools, Government departments (e.g., education, e-safety), famous influential role models, family/friends.
- Thinking about how a program could be delivered -
  - What platforms would you find best for a program delivery, Example probing questions website, apps, etc
  - How would a program be best delivered for use by you and your family. Example probing questions would you prefer a one-time delivery where you receive all the information at once and you could refer to it as needed OR a longer-term delivery with new information released weekly/fortnightly (e.g., 6-12 weeks) *new information would be a new topic such as being a positive role model, tips to providing active alternatives to screen.

Thinking about the information or strategies the program could contain to help you manage screen time?

- - What types of strategies would be useful to your family? For example: Information on the health risks of screen use; Family challenges to turn off screens; Device setting e.g., focus mode, restricted/reduced access; Monitoring tools to show your current screen use; Any others?
  - Would it be more feasible within your family if the program was designed for both parent and child or just child-focused?
  - Would you use interactive components of a program such as editable family media plans, goal setting and achievement boards, or would you prefer static content?

*We know that motivation and engagement in behaviour change programs often peaks early and then drops off.* What would help maintain your engagement in a screen time management program? *Example probing questions* would it help to receive reminder emails/texts to complete challenges or when new information has been released? How often would you like reminders?

Is there anything else you would like to mention about screen time in your home or what would help you to manage it?

Thanks for your time. *Stop recording*

**CHILD QUESTIONS**

- What are the main types of screens that you use at home?
- Is managing your screen time a challenge for you and your family? Yes/No, prompt: why do you think that?
- With COVID-19 lockdown restrictions, have your screen time behaviours changed?
- Do you have rules or strategies around screen use in your home? *Example probing questions:* no screens in the bedroom, usage/management controls? *Example probing questions* timers for use. Who gives you these rules/strategies?
- Does screen time impact how you and your family interact and communicates with each other (leads to arguments/ more or less talking with each other)?

We are wanting to develop ways to help families reduce screen time. We’d like to hear your input on this.

- Firstly, can you tell us who talks to you about screen time?

Outside of your parents, who else would listen to if they spoke to you about screen time rules and strategies? *Example probing questions* sports players, role models, teachers, Doctor, family/friends?

Now we have some questions about if your parents [who they mentioned talks about screen time] wanted to use new ways/strategies to reduce your screen time:

- Firstly, would you want to be involved in creating the ideas/strategies or would you rather your parents simply told you how, when and where to use your [device]/screens?
- Do you have any ideas of fun or interesting ways to help children your age reduce their screen time?

*Example probing questions:* reward charts, games or challenges, other ideas

- Would you want your parents/family to also use the new ways/strategies to reduce their screen time?
- Would you want your friends to also be involved in reducing their screen time?

We know that motivation and engagement in behaviour change programs often peaks early and then drops off, how could we keep you motivated, engaged and interested in a program that would run for a longer time (e.g. 16 weeks? 6 months)? *Example probing questions*

new challenges to help keep you off your devices? Being told about the risks of too much screen time? Incentives to do something (e.g. chores) to use your device, any other ideas?

- Is there anything else you would like to mention about screen time in your home or what would help you to reduce screen time?

Thanks for your time. *Stop recording*
