# Supplementary material for: “Everything kind of revolves around technology”: a qualitative exploration of families’ screen use experiences, and intervention suggestions
Source: BMC Public Health. 2022 Aug 23;22:1606. doi: 10.1186/s12889-022-14007-w (PMC9398049; doi:10.1186/s12889-022-14007-w)
Supplement: Supplementary file 2 — Additional file 2. Consolidated criteria for reporting qualitative studies (COREQ) checklist. [file 12889_2022_14007_MOESM2_ESM.docx]

Additional File 2: Consolidated criteria for reporting qualitative studies (COREQ) checklist

| Consolidated criteria for reporting qualitative studies (COREQ) checklist | | |  |
| --- | --- | --- | --- |
| **No** | **Item** | **Guide questions/description** |  |
| **Domain 1: Research team and reflexivity** | |  |  |
| Personal Characteristics | |  |  |
| 1. | Interviewer/facilitator | Which author/s conducted the interview or focus group? | LG |
| 2. | Credentials | What were the researcher's credentials? *E.g. PhD, MD* | Masters of Science (MSc) |
| 3. | Occupation | What was their occupation at the time of the study? | Research Assistant/Project Manager |
| 4. | Gender | Was the researcher male or female? | Female |
| 5. | Experience and training | What experience or training did the researcher have? | Previous qualitative and project experience |
| Relationship with participants | |  |  |
| 6. | Relationship established | Was a relationship established prior to study commencement? | No |
| 7. | Participant knowledge of the interviewer | What did the participants know about the researcher? e*.g. personal goals, reasons for doing the research* | Participants were aware the interviewer was employed to conduct the study and interviews |
| 8. | Interviewer characteristics | What characteristics were reported about the interviewer/facilitator? e.g. *Bias, assumptions, reasons and interests in the research topic* | Participants were aware the interviewer was employed to conduct the study and interviews |
| **Domain 2: study design** | |  |  |
| Theoretical framework | |  |  |
| 9. | Methodological orientation and Theory | What methodological orientation was stated to underpin the study? *e.g. grounded theory, discourse analysis, ethnography, phenomenology, content analysis* | Thematic analysis, content analysis |
| Participant selection | |  |  |
| 10. | Sampling | How were participants selected? *e.g. purposive, convenience, consecutive, snowball* | Snowball |
| 11. | Method of approach | How were participants approached? e*.g. face-to-face, telephone, mail, email* | Email |
| 12. | Sample size | How many participants were in the study? | 63 participants |
| 13. | Non-participation | How many people refused to participate or dropped out? Reasons? | Email invitations were sent to sixty-two families; 27 did not respond, 3 did not attend scheduled interview, one email “bounced” and one responded after the interviews were completed. |
| Setting | |  |  |
| 14. | Setting of data collection | Where was the data collected? e*.g. home, clinic, workplace* | Home |
| 15. | Presence of non-participants | Was anyone else present besides the participants and researchers? | No |
| 16. | Description of sample | What are the important characteristics of the sample? *e.g. demographic data, date* | Age, sex described |
| Data collection | |  |  |
| 17. | Interview guide | Were questions, prompts, guides provided by the authors? Was it pilot tested? | Yes |
| 18. | Repeat interviews | Were repeat interviews carried out? If yes, how many? | No |
| 19. | Audio/visual recording | Did the research use audio or visual recording to collect the data? | Yes, audio recorded |
| 20. | Field notes | Were field notes made during and/or after the interview or focus group? | Yes |
| 21. | Duration | What was the duration of the interviews or focus group? | Mean length 38 minutes, 13 seconds |
| 22. | Data saturation | Was data saturation discussed? | Yes, reached |
| 23. | Transcripts returned | Were transcripts returned to participants for comment and/or correction? | No |
| **Domain 3: analysis and findings** | |  |  |
| Data analysis | |  |  |
| 24. | Number of data coders | How many data coders coded the data? | Two (LA, LG) |
| 25. | Description of the coding tree | Did authors provide a description of the coding tree? | Yes, Table 1 |
| 26. | Derivation of themes | Were themes identified in advance or derived from the data? | Both |
| 27. | Software | What software, if applicable, was used to manage the data? | NVivo version 12.6 |
| 28. | Participant checking | Did participants provide feedback on the findings? | Yes, non-identifiable aggregated results were provided to participants. |
| Reporting |  |  |  |
| 29. | Quotations presented | Were participant quotations presented to illustrate the themes / findings? Was each quotation identified? e*.g. participant number* | Yes |
| 30. | Data and findings consistent | Was there consistency between the data presented and the findings? | Yes |
| 31. | Clarity of major themes | Were major themes clearly presented in the findings? | Yes |
| 32. | Clarity of minor themes | Is there a description of diverse cases or discussion of minor themes? | Yes |
